# *In vitro* Accuracy of Dental 3D Printers for the Fabrication of Laminate Veneers with Different Preparation Designs 

**DOI:** 10.30476/dentjods.2025.105367.2590

**Published:** 2025-12-01

**Authors:** Mohammad Amin Bafandeh, Mohammad Alihemmati, Ali Jamali Ghomi, Maryam Jahangiri, Yasaman Sherafatmand, Sayed Shojaedin Shayegh

**Affiliations:** 1 Dept. of Prosthodontics, Faculty of Dentistry, Shahed University, Tehran, Iran.; 2 Postgraduate Student, Dept. of Prosthodontics, Faculty of Dentistry, Shahed University, Tehran, Iran.; 3 PhD Candidate of Dental Biomaterials, Dept. of Dental Biomaterials, Faculty of Dentistry, Tehran University of Medical Sciences, Tehran, Iran.

**Keywords:** Dimensional Measurement Accuracy, Printing, Three-Dimensional, Dental Veneers

## Abstract

**Background:**

The adoption of three-dimensional (3D) printing in dentistry for prosthetic workflows is increasing. A crucial step in the indirect fabrication of laminate veneers involves creating accurate master casts from digital impressions. However, there is limited information available regarding the accuracy of dental 3D printers in fabricating these master casts when different tooth preparation designs for laminate veneers are employed.

**Purpose:**

This study aimed to assess and compare the accuracy (trueness and precision) of dental 3D printers in fabricating master casts for laminate veneers featuring three different incisal edge preparation designs (butt-joint, window, and palatal extension).

**Materials and Method:**

This *in vitro*, experimental study was conducted on three dental models made of polyether ether ketone (PEEK) with central incisor and canine teeth with three incisal preparation designs of window, butt-joint, and palatal extension for fabrication of laminate veneers. The models were scanned by the same laboratory scanner, and the standard tessellation language (STL) files were printed by four printers: Prodent (material jetting [MJ]), Asiga (digital light processing [DLP]), Hunter (DLP), and Luminous (light-emitting diode [LED]), 30 times. A total of 120 printed models were scanned again, and their scan files in STL format were compared with the reference model file to assess the trueness and precision of the printers. Data were analyzed using paired and independent t-tests, one-way analysis of variance (ANOVA), and Tukey test (α= 0.05).

**Results:**

Asiga printer showed significantly higher trueness and precision than other printers (*p*< 0.05). No significant difference was found among other printers in trueness or precision (*p*> 0.05). The precision of window preparation design was significantly lower than other preparation designs (*p*< 0.05). No significant difference was found among other preparation designs in precision (*p*> 0.05). The difference in trueness was not significant among the preparation designs (*p*> 0.05).

**Conclusion:**

Asiga printer showed significantly higher trueness and precision than other tested printers for fabrication of laminate veneers. Also, window preparation of the incisal edge resulted in significantly lower precision than butt-joint and palatal extension designs.

## Introduction

By the advances in digital technology, digital dentistry is gaining increasing popularity [ 1 - 2 ]. Studies assessing the accuracy of digital models compared to the conventional models can be categorized into three groups methodologically: (I) those addressing errors in linear measurements, (II) those addressing errors two-dimensionally and three-dimensionally, and (III) those assessing the passive fit of supra-structures on different models [ 3 ].

The *in vitro* assessments of measurement accuracy in dental research are typically based on the concepts of trueness and precision, which are widely accepted in metrology literature. Trueness refers to the closeness of a measurement to the actual or reference value, while precision indicates the consistency or repeatability of measurements. These parameters are often evaluated using statistical tools such as the root mean square (RMS) to assess deviations from a reference model. In this context, 1 RMS is equivalent to 100 µm. Random errors in each measurement technique can influence precision [ 4 ]. Digital dental technology aims to provide easier and faster solutions compared to the conventional methods and yield more accurate results at a lower cost [ 1 , 5 - 7 ]. Digital dental technology is also favored by dental students [ 7-8 ].

Duplication of a precise model of dental arch is imperative for the fabrication of prosthetic restorations [ 9 ]. Dental impressions are conventionally used for this purpose, and it has been well confirmed that accuracy of impressions can determine the accuracy of restoration to a great extent [ 10 - 11 ]. 

Digital oral impressions and computer-aided design/ computer-aided manufacturing (CAD/CAM) of dental restorations date back to 1980 [ 10 - 13 ]. CAD/ CAM technology is a developing field in digital dentistry. Dental restorations can be fabricated by two techniques: direct and indirect [ 14 ]. In the indirect approach, a conventional impression is made from the dental arch preferably by using silicone impression material, and a dental cast is fabricated. The cast is then scanned by a laboratory scanner and digital processing is initiated. In the direct method, however, an intraoral scanner is used to scan the teeth intraorally and make a digital impression [ 15 ]. The advantages of the latter technique include personalization of restoration by expert technicians and the ability to use more durable materials for the restoration since the restoration is fabricated by milling [ 8 ]. Physical cast is required to ensure precise proximal and occlusal contacts in this method [ 10 - 12 ].

At present, definitive casts can be fabricated by subtractive or additive technology. Additive manufacturing, also known as 3D printing, is defined as layer-by-layer application of material to fabricate an object from data of a 3D model [ 16 - 17 ]. 

Precision and trueness of 3D printers are among the most influential factors on the accuracy of final restoration. Considering the availability of different types of 3D printers in the market, this study aimed to assess and compare the accuracy of dental 3D printers for the fabrication of laminate veneers with different preparation designs. The null hypothesis of the study was that no significant difference would be found in precision and trueness of different printers in different preparation designs. 

## Materials and Method

In this *in vitro*, experimental study, central incisor and canine teeth of one quadrant of acrylic models of maxilla (500A, Nissin Dental Products Inc, Japan) received a laminate veneer preparation with three different designs for the incisal edge: palatal extension, window, and butt- joint. 

**Figure 1:**
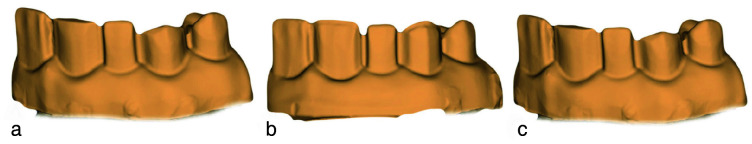
Scan files of the three incisal edge preparation designs, **a:** Butt joint, **b:** Window, **c:** Palatal extension

**Figure 2:**
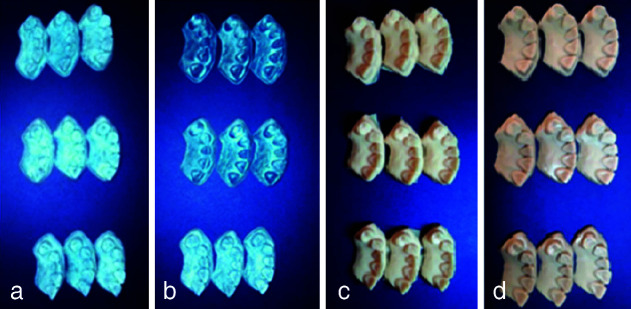
The models printed by the printers in order from left to right are, **a:** Luminous, **b:** Prodent, **c:** Asiga, d: Hunter

**Figure 3:**
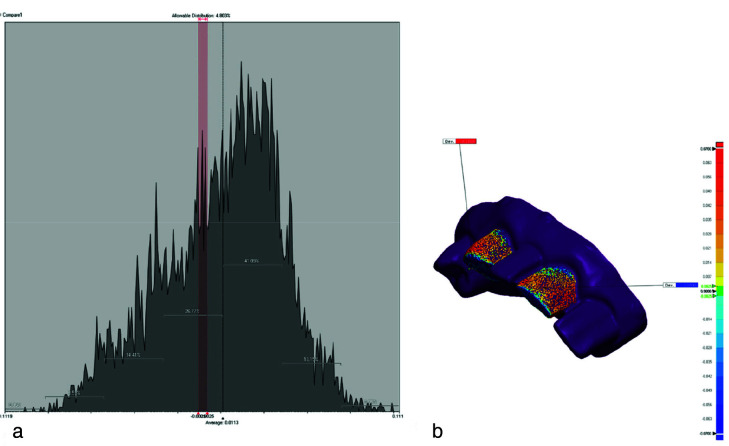
A representative sample of the analyses conducted in Geomagic software, **a:** Data distribution graph; **b:** color scheme range of comparison

A laboratory scanner (Open Technologies, Italy) was then used to scan the models with white light to generate three STL files, which were used for the milling of reference master casts using Inlab mc ×5 unit (Dentsply Sirona, Charlotte, North Carolina, USA) milling machine. The material used for the milling of the casts was polyether ether ketone (PEEK) due to its hardness and higher scan ability. The models of this study included three master dies made of PEEK [ 18 ]. A digital reference file was created by scanning the cast using an optical 3D scanner (ATOS Core, GOM, Germany) with a trueness and precision of 2 µm as declared by the manufacturer. A laboratory scanner (Open Technologies, Italy) was then used to scan the three master models made of PEEK (breCAM Bio HPP blank; Bredent, Senden, Germany, LOT 381115) (Figure 1). Next, four 3D printers namely Prodent/MJ=material jetting (Bonyan Mechatronic Iranian Co. Tabriz, Iran), Luminous/ LED= light-emitting diode (Bonyan Mechatronic Iranian Co. Tabriz, Iran), Asiga /DLP = Digital Light Processing (Asiga Co. Alexandria, Australia), and Hunter/ DLP= Digital Light Processing (Flashforge 3D Technology Co. Zhejiang, China) were used to print 30 samples (10 from each preparation design) with 0-degree angle relative to the printer plate. We selected these four printers based on their availability (Figure 2). Also, we chose two DLP printers to compare different companies with the same mechanism. In a 0-degree angle, the layers are applied perpendicular to the longitudinal axis of each restoration. The models were fabricated at the center of the plate with 25-µm layer thickness. Three repetitions were also performed, yielding 120 samples. All printers were calibrated by a technician prior to use. After completion of 3D printing, post-processing was performed according to the resin manufacturer’s instructions. The entire scanning and printing process was performed by the same operator according to the best protocol recommended by the manufacturer. A laboratory scanner (Open Technologies, Italy) was then used to scan all the 120 samples. The STL files were directly transferred from the scanner to Geomagic Design X software (Geomagic Control X, version 2018. 1.1; 3D Systems, USA). The STL files were then individually superimposed on the STL file of the reference scanner such that the reference STL file was considered as CAD file, and the test STL files were superimposed on it as mesh using the best-fit protocol according to the teeth and cylindrical index, and a final file was generated for the purpose of comparison. The Edit Boundaries feature was used to crop the excess margins including the tissues and cylindrical index. The lines were then smoothened and refined. Next, Geomagic Control X software (Geomagic Control X, version 2018 .1.1; 3D Systems, USA), which is a reverse engineering software, was used to measure the differences in median, mean, maximum and minimum shape and curvature in the final files (Figure 3). The range of differences of specimens was considered from -0.07 to +0.07µm. 

For calculation of precision, we randomly selected a scan and compared the others to it. Comparisons were made pairwised, between the reference file (which was the scan file #1) and the other two scans (different prepared veneer designs). To calculate the trueness of the three 3D-printers, the model scans of each group were compared to the STL file of the reference model.

### Sample size

The required sample size was calculated to be 8.3 for each printer assuming 95% confidence interval (alpha= 0.05), standard error of 0.05, and variance of 40µm. Thus, 4 printers printed 3 preparation designs 30 times, yielding a total of 120 samples.

### Statistical analysis

The normality of data distribution was confirmed by the Shapiro-Wilk test (*p*> 0.05). Thus, the groups were compared by one-way ANOVA followed by pairwise comparisons with the Tukey test. An independent t-test was used to compare the 3D printers, and paired t-test was applied to compare the incisal edge preparation designs. All statistical analyses were performed by SPSS version 22 (SPSS Inc., IL, USA) at 0.05 level of significance.

## Results

### Trueness based on preparation design

Table 1 presents the measures of central dispersion for trueness of all four 3D printers for the three types of incisal edge preparation designs. 

**Table 1:** Mean trueness of printers by preparation design

Preparation Design	Mean Trueness Asiga	Mean Trueness Hunter	Mean Trueness Prodent	Mean Trueness Luminous
Butt-joint	0.0099	0.0212	0.0211	0.0222
Window	0.0099	0.0137	0.0138	0.0301
Palatal extension	0.0089	0.0102	0.0255	0.0295

In the butt-joint design, Hunter had the lowest and Asiga had the highest trueness. In the window design, Hunter had the lowest and Asiga had the highest trueness. In the palatal extension design, Hunter had the lowest and Asiga had the highest trueness. 

### Precision based on preparation design

Table 2 presents the measures of central dispersion for precision of all four 3D printers for the three types of incisal edge preparation designs. In the butt-joint design, Hunter had the lowest and Asiga had the highest precision.

**Table 2:** Mean precision of printers by preparation design

Preparation Design	Mean Precision Asiga	Mean Precision Hunter	Mean Precision Prodent	Mean Precision Luminous
Butt-joint	0.0043	0.0159	0.0039	0.0032
Window	0.0096	0.0187	0.0021	0.0161
Palatal extension	0.0041	0.0015	0.0244	0.0106

In the window design, Hunter had the lowest and Asiga had the highest precision.

In the palatal extension design, Hunter had the lowest and Asiga had the highest precision.

### Precision and trueness of the printers in all three designs

As shown in Table 3, the lowest RMS and variance and the highest precision belonged to Asiga, and the lowest mean precision belonged to Luminous. Also, the lowest RMS and variance and the highest trueness belonged to Asiga, and the lowest trueness belonged to Hunter. 

**Table 3:** Precision and trueness of printers across three preparation designs

Parameter	Printer	Butt Joint Mean	Window Mean	Palatal Extension Mean
Precision	Luminous	0.0293	0.0749	0.0661
	Prodent	0.022	0.0229	0.0597
	Hunter	0.0465	0.0445	0.0382
	Asiga	0.016	0.0225	0.0192
Trueness	Luminous	0.0661	0.0742	0.0742
	Prodent	0.0597	0.044	0.044
	Hunter	0.0382	0.0618	0.0618
	Asiga	0.0192	0.0279	0.0279

### Trueness based on type of printer

In Asiga, the lowest trueness (due to high RMS) belong-ed to the butt-joint design and the highest trueness (due to low RMS) belonged to the palatal extension design.

In Hunter, the lowest trueness belonged to the palatal extension design and the highest trueness belonged to the butt joint design.

In Prodent, the lowest trueness belonged to the palatal extension design and the highest trueness belonged to the window design. In Luminous, the lowest trueness belonged to the window design and the highest trueness belonged to the butt-joint design. In total, the palatal extension design was the lowest and the butt-joint design had the highest trueness of all four printers. 

### Precision based on type of printer 

In Asiga, the lowest precision belonged to the window design and the highest to the butt-joint design. 

In Hunter, the lowest precision belonged to the butt-joint design and the highest to the palatal extension design. In Prodent, the lowest precision belonged to the palatal extension design and the highest to the butt-joint design. In Luminous, the lowest precision belonged to the palatal extension design and the highest to the butt-joint design. 

In total, the lowest precision belonged to the palatal extension and the highest to the butt-joint design. 

### Analytical comparison of precision of printers for each preparation design

As shown in Table 3, a significant difference existed in precision of the printers in window design (*p*= 0.012) such that Asiga had the highest and Hunter had the lowest precision.

However, no significant difference existed in the mean precision of the four printers in the butt-joint, and palatal extension designs (*p*> 0.05). Pairwise comparisons of printers showed significantly higher precision of Asiga than all other printers (*p*< 0.05). No significant difference was found between other printers (*p*> 0.05).

### Analytical comparison of trueness of printers for each preparation design

As shown in Table 3, no significant difference existed in the mean trueness of the four printers in any preparation design (*p*> 0.05). Pairwise comparisons of printers showed significantly higher trueness of Asiga than all other printers (*p*< 0.05). No significant difference was found between other printers (*p*> 0.05). 

## Discussion

This study assessed and compared the accuracy of dental 3D printers for the fabrication of laminate veneers with different preparation designs. The results showed significantly higher trueness and precision of Asiga than other printers. However, the difference in this regard between other printers was not significant. The precision of the window preparation design was significantly lower than other designs. However, no significant difference was found between other preparation designs. So, it is not recommended to use printed cast for window preparation designs. The trueness of different designs was similar too. Thus, the null hypothesis of the study was rejected.

Previous research has revealed conflicting outcomes. While some researchers found no effect [ 19 - 20 ], others concluded that more surface area and complexity in the preparation design reduced scanning accuracy [ 21 - 22 ].

Consistent with the present results, Papaspyridakos *et al*. [ 23 ] showed that the Asiga Max UV printer yielded the lowest mean error. Nestler *et al*. [ 24 ] assessed the accuracy of casts printed by different printers and reported that Asiga MAX UV had the highest accuracy although both extrusion-based and photopolymerization- based printers were accurate. Ishida and Miyasaka [ 25 ] and Etemad-Shahidi *et al*. [ 26 ] reported higher performance of printers with digital light processing technology than other printers. Similarly, the Asiga printer with digital light processing technology showed higher accuracy than other printers in the present study. The distinguishing point of our study from others is the investigation of the higher accuracy of the Asiga in different veneer designs. Also, Etemad-Shahidi *et al*. [ 26 ] showed that the difference in accuracy among different printers was < 500 µm, which was different from the present study showing a maximum discrepancy of 700 µm. Also, Anna Németh *et al*. [ 27 ] compared the accuracy of 3D printed full-arch dental models manufactured using seven printing techniques (SLA, DLP, fused deposition modeling/fused filament fabrication (FDM/FFF), MultiJet (MJ), PJ, continuous liquid interface production (CLIP), and LCD technology). This network meta-analysis showed that SLA, DLP, and PJ Technologies are the most accurate printing techniques. Francois Rouzé l'Alzit *et al*. [ 28 ] compared the precision and trueness of two different surgical guides (small and large extent) with five printers (SLA, DLP, FDM, SLS, Inkjet). SLA, DLP, and PJ Technologies were the most accurate printing techniques. Unlike this study, Hazem Yousef *et al*. [ 29 ] measured the accuracy of Asiga MAX and ProJet 3510 DPPro printers. The MJ-printed cast (ProJet 3510 DPPro) were more accurate than the DLP-printed cast. Since MJ polymerized the resin with UV light, the 3D-printed object is exact, and the printing layer thickness can be under 20 μm; no surface finishing is required [ 30 ]. Yi-Cheng Lai *et al*. [ 31 ] examined the accuracy of 4 printers, including a DLP 3D printer, an LED 3D printer, a CLIP 3D printer, and an SLA 3D printer for two finish lines. This study reported the opposite result and showed that the highest accuracy was related to the CLIP and SLA 3D printers. The accuracy of printers in three different preparation designs was evaluated in this study, which was an advantage because Tian *et al*. [ 32 ] showed higher accuracy of printers on flat surfaces. Thus, preparation design can affect the accuracy of printers. In this study, the precision of the window preparation design was significantly lower than other preparation designs (*p*< 0.05) but there was no significant difference among other preparation designs in precision (*p*> 0.05). Also, l'Alzit *et al*. [ 28 ] compared the precision and trueness of small and large extended surgical guides with five printers (SLA, DLP, FDM, SLS, Inkjet). There were significant differences between small-extent and large-extent guides. Overall, printing small areas with FDM and Inkjet printers is more accurate. However, SLA, DLP, and PJ Technologies showed similar results in terms of trueness and precision for both groups. On the other hand, Yi-Cheng Lai *et al*. [ 31 ] investigated the precision of shoulder and chamfer finish lines. They reported no significant effects from different finish line designs on the accuracy of printed casts. The accuracy of printers in storage conditions and at different times was not evaluated in this study, which was a disadvantage, However, Hazem Yousef *et al*. [ 29 ] stated that exposure of Asiga MAX prints to light within 3 months will change their color and reduce their accuracy. Yi-Cheng Lai *et al*. [ 31 ] investigated the accuracy of 4 printers in different storage conditions (exposure to light and darkness) at different times (within 36 hours, 1 month, and 3 months). This study reported that keeping the printed casts for more than a month and exposing them to light alters the accuracy of the prints. 

One notable limitation of this *in vitro* study is the evaluation of accuracy using only four specific 3D printer models. The diverse range of 3D printing technologies and available printers in the market suggests that the findings, particularly regarding the superior performance of the Asiga printer, might not be generalizable to all other systems. Furthermore, the study did not assess the potential impact of storage conditions (e.g., light exposure, humidity) or the duration of storage on the accuracy of the printed veneers over time, factors that could influence the long-term clinical performance of restorations.

This study provides valuable insights into the accuracy of specific dental 3D printers for fabricating laminate veneers with varying incisal edge preparation designs. These results contribute to the growing body of evidence regarding the capabilities of different 3D printing technologies in dentistry, aligning with some prior research indicating the high accuracy of DLP-based systems like the Asiga. However, the conflicting findings in the literature underscore the need for continued investigation across a broader range of printers and preparation designs. Clinically, our findings suggest that while DLP printers, particularly the Asiga model in this study, hold promise for accurate veneer fabrication, careful consideration should be given to the preparation design, with caution advised against relying on printed casts for window preparations. Future research should explore the accuracy of a wider array of 3D printers, different printing materials, and the impact of post-processing and storage conditions to provide a more comprehensive understanding of their clinical applicability in restorative dentistry. 

## Conclusion

Our findings demonstrated that the Asiga printer exhibited significantly superior trueness and precision compared to the other tested models (Prodent, Luminous, and Hunter). Furthermore, the window preparation design was associated with a significantly lower precision compared to the butt-joint and palatal extension designs, suggesting that printed casts may not be the optimal approach for this specific preparation type. While the trueness across different preparation designs was comparable, the observed discrepancies in precision highlight the influence of preparation geometry on the final accuracy of printed restorations. 
